# Astrocyte Hypertrophy Contributes to Aberrant Neurogenesis after Traumatic Brain Injury

**DOI:** 10.1155/2016/1347987

**Published:** 2016-05-04

**Authors:** Clark Robinson, Christopher Apgar, Lee A. Shapiro

**Affiliations:** ^1^Department of Surgery, Texas A&M University Health Science Center, College of Medicine, Temple, TX 76504, USA; ^2^Central Texas Veterans Health Care System, Temple, TX 76504, USA; ^3^Department of Neurosurgery, Neuroscience Research Institute, Scott & White Hospital, Temple, TX 76508, USA

## Abstract

Traumatic brain injury (TBI) is a widespread epidemic with severe cognitive, affective, and behavioral consequences. TBIs typically result in a relatively rapid inflammatory and neuroinflammatory response. A major component of the neuroinflammatory response is astrocytes, a type of glial cell in the brain. Astrocytes are important in maintaining the integrity of neuronal functioning, and it is possible that astrocyte hypertrophy after TBIs might contribute to pathogenesis. The hippocampus is a unique brain region, because neurogenesis persists in adults. Accumulating evidence supports the functional importance of these newborn neurons and their associated astrocytes. Alterations to either of these cell types can influence neuronal functioning. To determine if hypertrophied astrocytes might negatively influence immature neurons in the dentate gyrus, astrocyte and newborn neurons were analyzed at 30 days following a TBI in mice. The results demonstrate a loss of radial glial-like processes extending through the granule cell layer after TBI, as well as ectopic growth and migration of immature dentate neurons. The results further show newborn neurons in close association with hypertrophied astrocytes, suggesting a role for the astrocytes in aberrant neurogenesis. Future studies are needed to determine the functional significance of these alterations to the astrocyte/immature neurons after TBI.

## 1. Introduction

Traumatic brain injuries (TBIs) have multiple causes, from motor vehicle accidents, falls, sports-related injuries, violence, and military activity, making TBI a serious health concern around the world. The World Health Organization has stated that the incidence and long-term consequences of TBIs will continue to increase over the next five years, representing a continual increase to this already existing major cause of death and disability [1]. Not only can TBI result in a patient's loss of function, but, in some cases, TBI may progress into early dementia, depression, personality changes, and posttraumatic epilepsy [2]. Therefore, understanding the complex cascades of biological consequences is vital to improving diagnostic and treatment options.

Similar to a typical response against any tissue damage, TBI induces inflammatory and neuroinflammatory processes [2–4]. Astrocytes and microglial cells are key central nervous system components of the neuroinflammatory response. Following a TBI, these glial cells become activated, resulting in the release of proinflammatory molecules such as interleukin 1 beta (IL-1*β*), interleukin 6 (IL-6), macrophage inflammatory protein (MIP), and CCL2 [5–10]. One method to discern activated astrocytes is by their morphology and GFAP expression [11, 12].

Amongst the neuroinflammatory consequences of astrocyte activation, previous studies have indicated that astrocyte hypertrophy can provide an anatomical substrate for the aberrant growth of newborn dentate granule cells [13–15]. More specifically, Shapiro et al. [13] demonstrated that because the glial cells at the border between the hilus and granule cell layer were closely apposed to the DCX+ processes, the latter processes grow into the hilus along an ectopic glial scaffold, where astrocytic processes preferentially orient into the hilus, rather than through the granule cell layer. This aberration is different than in the normal adult rodent brain, where GFAP-expressing radial glial-like cells give birth to and wrap around new born dentate granule cells in a one-to-one relationship to nurture and guide the growth and integration of the newborn neurons. As part of this relationship, the radial glial cells extend a process that extends radially through the granule cell layer and the growing apical dendrite from the newborn neuron extends along this radial glial process. Thus, the radial glial-like cell helps to guide the newborn neurons as they migrate from the subgranular zone and grow into the granule cell layer [16, 17]. In addition to the growing apical dendrite, newborn neurons in the normal adult rodent brain often sprout a basal dendrite early in their development. In the normal rodent brain, after about 72 hours, this transient basal dendrite from the newborn neuron will begin to retract or curve back such that they course parallel to the granule cell layer [18]. However, following brain insults such as epileptogenic stimulus, neurogenesis is known to be altered, as is the relationship between newborn neurons and radial glial-like astrocytes [14–16].

Several studies have demonstrated a link between astrocyte activation and aberrant neurogenesis [14, 19–22]. These alterations to neurogenesis include increased and/or decreased numbers of newborn neurons and altered survival, migration, and integration of the newborn neurons into existing brain circuitry. For example, following TBIs or seizures, neurogenesis is typically increased in a transient fashion, such that, at short durations after injury or insult (<30 days after insult), subventricular zone and hippocampal neurogenesis is increased, whereas at >30 days after injury, a decrease in the number of newborn neurons is observed [23–25]. In addition, newborn neurons in the hippocampus have been observed to ectopically migrate into the hilus following seizures, and these ectopic cells contribute to aberrant hippocampal function [26, 27]. The growth and integration of newborn neurons are also observed after seizures, such that the newborn granule cells were observed to sprout basal dendrites that course deep into the hilus, where they are synaptically targeted by mossy fibers from granule cells [28–30]. This granule cell to granule cell connectivity constitutes a recurrent circuitry, and computational models as well as electrophysiological studies have demonstrated that such a circuit significantly disrupts normal hippocampal functioning [31, 32].

Considering the importance of hippocampal function to a wide variety of behavioral, emotional, reproductive, and cognitive functions, it is possible that alterations to hippocampal neurogenesis can negatively impact hippocampal function, leading to functional deficits that include disorders of learning and memory, affective behaviors, and cognitive dysfunction. Indeed, these types of short- and long-term deficits are often seen after TBI in humans [33–35] and in animal models [36, 37]. Thus, it is possible that changes to newborn neurons and their associated astrocytes might occur following a TBI, thereby contributing to functional deficits.

Previous studies have shown that cortical astrocytes are activated soon after a TBI [5, 6]. Other studies have demonstrated altered neurogenesis and activated astrocytes in the hippocampus at various time points and in various TBI animal models, as well as in clinical cases [38–40]. Given these data, we hypothesize that TBI causes astrocyte and neurogenic alterations in the hippocampus, such that the astrocytic changes provide an anatomical substrate for the aberrant growth of hilar basal dendrites from these newborn neurons. To test this hypothesis, we examined astrocytes, newborn neurons, and the relationship between these cell types in the hippocampus at 30 days following a TBI in mice.

## 2. Methods

### 2.1. Fluid Percussion Injury (FPI)

Mice were randomly separated into two cohorts—a sham group (*N* = 5) and an FPI group (*N* = 5). Prior to sham or FPI procedures, all mice were anesthetized with an initial dose of 4% isoflurane and oxygen for anesthesia induction and maintained anesthesia on 2% isoflurane. Sham and FPI were performed as previously described [8, 41]. Briefly, once the mice were fully anesthetized, their heads were shaved. Strict sterile technique was followed throughout all surgical procedures. All animals were placed in a stereotaxic instrument with an attachment for mouse surgery (Stoelting, Inc., IL, USA). A 2 mm hole was drilled into the skull over the left parietal cortex, making sure to leave the dura intact. A female luer-lock (PlasticOne) was connected to the hole in the skull and mice in the FPI group received a pressure pulse of 1.5–1.7 atm from the FPI apparatus through the luer-lock for 12–16 ms. Sham animals received no pressure pulse while connected to the apparatus. Animals were housed singly after FPI with a 12-hour light-dark cycle (light on 6:00 and light off 18:00). All animals had continuous access to food and water.

### 2.2. Tissue Preparation

30 days after surgery, mice in both groups were sacrificed and fixed under anesthesia by transcardial perfusion as previously described [6, 8]. Briefly, a peristaltic pump was used to deliver 0.9% saline through the left ventricle until the runoff through the cut right atrium ran clear. This was followed by pumping 4% paraformaldehyde through the left ventricle. The brains were allowed to postfix for 24 hrs in the skull, after which they were extracted and fixed for 24 hours in 4% paraformaldehyde and subsequently cut into 50-micrometer-thick serial coronal sections with a vibratome.

### 2.3. Doublecortin (DCX) Immunohistochemistry

Immature neurons were immunohistochemically stained for DCX as previously described [14, 16, 23]. Incubation of the primary antibody was done on tissue slices (*N* = 4 slices per mouse for each group) incubated in the dark, rotating at room temperature, for 24 hours, in PBS containing 0.005% tween, 5% normal horse serum, and DCX antibodies to the N and C termini (1 : 500 each, Santa Cruz Biotech). After staining, the tissue slices were rinsed 3 times for 5 minutes each in 0.01 M PBS. The tissue slices were next incubated for 90 minutes, rotating in the dark, at room temperature in PBS containing 0.005% tween, 5% normal horse serum, and fluorescently tagged donkey anti-goat antibody (1 : 200; Alexa Flour 488; Invitrogen). After 90 minutes, the tissue slices were rinsed 3 times for 5 minutes each in 0.01 M PBS, before being mounted onto glass slides. Slides were allowed to dry overnight and cover slips were applied with Vectashield Hardset (Vector labs, Burlingame, CA). The slides were coded for subsequent analysis by reviewers blind to the condition of the tissue.

### 2.4. Glial Fibrillary Acidic Protein (GFAP) Immunohistochemistry

Astrocytes and radial glial-like astrocytes were labeled as previously described [13]. Briefly, tissue slices (*N* = 4-5 slices per mouse for each group) were incubated in the dark, at room temperature, for 24 hours in a mixture of 0.01 M PBS, 3% normal goat serum, and CY3 fluorescently tagged, primary GFAP antibody (1 : 1000; Sigma). After staining, the tissue slices were washed 3 times in 0.01 M PBS, mounted onto glass slides, and cover slips were applied with Vectashield Hardset (Vector labs, Burlingame, CA). As above, the slides were coded to eliminate potential reviewer bias.

### 2.5. Quantification of DCX-Labeled Cells in the Dentate Gyrus

**Figure 1:**
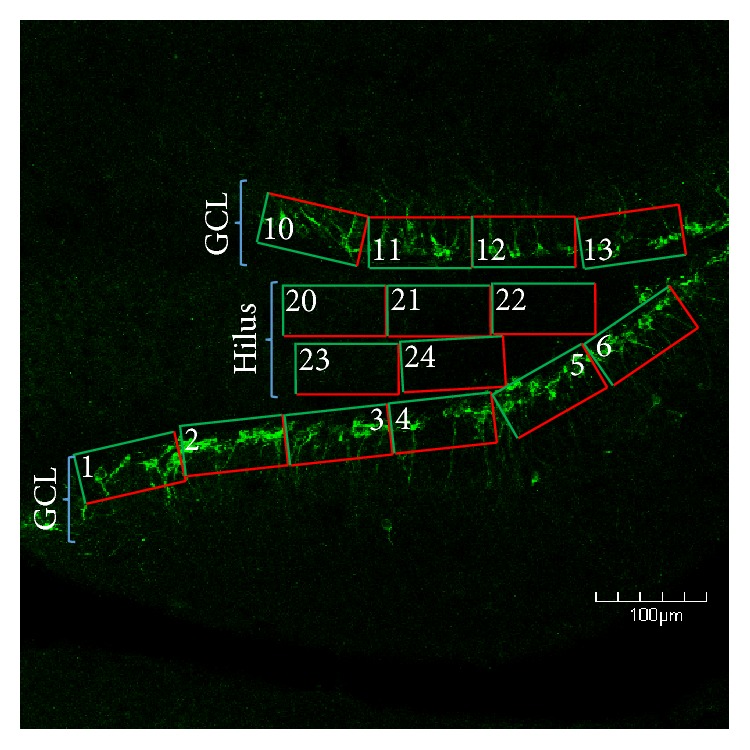
Confocal micrograph depicting the grid layout used to randomly select areas of the dentate gyrus in which newborn neurons were quantified. For each region of interest, we overlaid a series of numbered boxes. We then set the range of numbers in a random number generator and had it choose which grid numbers would be quantified at higher magnification. In this schema, the hilus and supra- and infrapyramidal blades of the granule cell layer (GCL) were quantified, in order to sample the entire dentate gyrus. Note that green portions of the grids are inclusion lines and red portions are exclusion lines. The grids along the GCL were positioned to include cells in the subgranular zone to the upper 3/5ths of the granule cell layer. The grids in the hilus were positioned to exclude cells in the subgranular zone, as DCX+ cells in this location are not necessarily hilar ectopic granule cells, and GFAP+ cells in this region typically display a morphology that is distinctly different than those in the hilus. It is pertinent to note that the apex and subjacent hilar areas are excluded from counting because cells in this region receive signals and cues from both the supra- and infrapyramidal blades of GCL. Scale bar = 100 *µ*m.

A series of images from the ipsi- and contralateral dentate gyri were captured on the Olympus IX-81 laser scanning confocal microscope. These sections spanned the rostral-caudal extent of the hippocampus from −1.34 to −2.54 from bregma. Equal sampling of the hippocampi within these coordinates was ensured by an investigator separate from the blinded raters (e.g., the coordinate for each slice was identified by an independent reviewer who did not partake in the image analysis). Once the images were captured, they were loaded into PowerPoint (Microsoft 2010) and grids were superimposed over the images to allow random selection of the boxes for subsequent quantification at higher magnification. Cellular and neuropil landmarks were used in the low magnification images to ensure that the borders were accurately observed during high magnification counting. Upon loading into PowerPoint, the images were coded a second time, and a different rater traced the borders of the hilus, the borders of the suprapyramidal subgranular zone extending into the first 4 layers of granule cells, and the borders of the infrapyramidal subgranular zone extending into the first 4 layers of granule cells (Figure 1). Per stereological guidelines, a grid containing an array of numbered 5000 *µ*m^2^ boxes was overlaid upon the traced regions of interest (Figure 1). Areas to be counted were identified using a random number generator to select the boxes in which counting would be performed (https://www.random.org/integers/). The number of boxes to be counted was predetermined based on stereological estimates. In most cases, 3 boxes from each region of interest were randomly selected for analysis using the random number generator. However, in the most rostral slices (−1.34 to 1.55 from bregma), only 2 boxes were selected because of the decreased size of the granule cell layer and hilus. Alternatively, in the most caudal sections (−2.28 to 2.54 from bregma), 4 boxes were randomly selected because of the increased size of the granule cell layer and hilus. Within each box, 1 lateral and 1 horizontal border were chosen as inclusion lines, and the other two borders were exclusion lines. This schema was consistently maintained for all slices. All cells within the box, or touching the inclusion lines, were counted, whereas cells contacting the exclusion lines were omitted.

### 2.6. Radial Glial Process Counting

Images from the infra- and suprapyramidal cell layers of the left and right side of each slice were captured using the same blinding method as above for DCX counting. Areas for analysis were determined by drawing boundaries around the suprapyramidal and infrapyramidal granule cell layers. The area of each granule cell layer was calculated using Image J (NIH v.1.49) and multiplied by the section thickness, as determined using the confocal microscope. All primary processes were counted throughout the entire traced granule cell layer by an experimenter blinded to the animal's condition. Secondary and tertiary branches were counted separately if they arborized within the upper 3/5ths of the granule cell layer. Processes that branched deeper than this were not counted separately. The origin of these processes was not considered, as the cell body was often outside of the plane of focus and the perikaryal cytoplasm does not always robustly stain with GFAP. The data were then divided by the image's calculated volume to obtain the number of processes per cubic micron:

(1) $$\begin{array}{c} \frac{\text{number of radial glial processes}}{\left(\text{area of region of interest}\times \text{depth of image series}\right)}. \end{array}$$

### 2.7. Densitometry

In order to assess astrocyte hypertrophy in the hilus, we used a derivation of a previous densitometry protocol [42] to quantify the intensity of pixels using Image J (NIH v.1.49). *Z*-stack image series of 18 microns deep were captured on the confocal microscope, starting from the first visible astrocytic process at the top of the image for the right and left hilus, for all slices. All *Z*-stack images were captured from coded slides, by an experimenter blinded to the condition of the animal. For image analysis, the *Z*-stacks were merged into a single image (Olympus V.3.2) and thresholded into a binary image using Image J (NIH v.1.49). The autothreshold function was used for this portion of the analysis. As above, a second blinded reviewer randomly placed grids throughout these regions and used the Image J software (NIH v.1.49) to measure the mean grey values of the binary images. It should be noted that GFAP+ cells at the border of the subgranular zone were excluded from analysis. The reason for this is that these cells typically have a very different appearance compared to cells within the hilus, because many of the cells at the subgranular zone border are progenitor (neuronal and/or astrocytic) cells in varying stages in the cell cycle. As such, these cells often appear vastly different than the hilar astrocytes, most of which lack the capabilities to give rise to newborn neurons.

### 2.8. Statistics

For all analysis, a SPSS was used to run Student's *t*-test to compare FPI and sham groups. Data are reported as means with error bars representing standard error of the mean (±SEM).

## 3. Results

### 3.1. DCX

**Figure 2:**
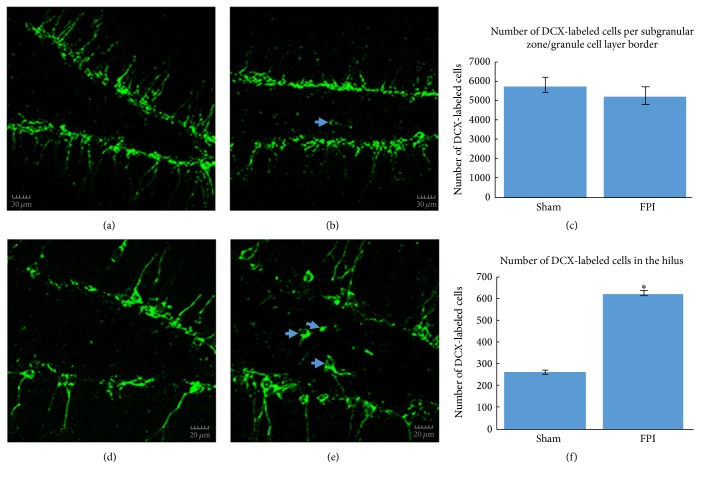
DCX-labeled immature neurons in the dentate gyrus. In (a), a relatively low magnification confocal micrograph is shown to depict both the infrapyramidal and suprapyramidal blades, as well as the hilus from a sham mouse. In (b), a fluorescent micrograph from an FPI mouse illustrates that, despite no significant differences in the number of DCX-labeled cells, the overall appearance of the DCX-labeled cells in the dentate gyrus is altered, as can be seen by the hilar ectopic granule cell (arrow). In (c), quantitative analysis of DCX-labeled cells at the subgranular zone/granule cell layer border revealed no significant differences between sham mice and FPI mice at 30 days after injury. In (d), a higher magnification confocal micrograph is shown to illustrate the typical appearance of granule cells in the dentate gyrus in a sham mouse. In (e), the confocal micrograph at higher magnification from an FPI mouse shows a relatively robust number of DCX-labeled hilar ectopic granule cells (arrows) at 30 days after a TBI. Such cells have been implicated in proepileptogenic circuits. In (f), quantitative analysis of the number of DCX-labeled hilar ectopic cells revealed a significant increase (^*∗*^ *P* < 0.009) in FPI mice compared to sham mice. Scale bars in (a) & (b) = 30 *µ*m and in (d) & (e) = 20 *µ*m.

The appearance of DCX-labeled cells in the dentate gyrus of sham mice was relatively normal, whereas in FPI mice, the morphology and location of some of these cells appeared altered (Figures 2(a), 2(b), 2(d), and 2(e)). Analysis of the number of DCX-labeled cells in this region revealed no significant difference between the sham mice and the FPI mice (*t* = 0.645; *P* = 0.441,  NS; Figure 2(c)). Conversely, there was a significant increase in the number of immature granule cells in the hilus of FPI mice compared to sham mice (*t* = 10.961; *P* < 0.009; Figure 2(f)).

### 3.2. Radial Glial-Like Processes

**Figure 3:**
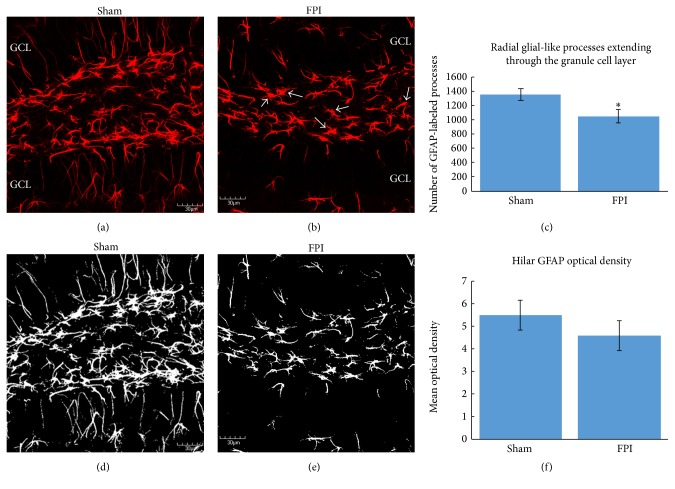
GFAP-labeled cells in the dentate gyrus. In (a), a confocal micrograph from a sham mouse is shown to depict the normal appearance of a robust number of radial glial processes extending through the granule cell layer (GCL). In (b), a representative confocal micrograph of an FPI mouse shows a decrease in the number of radial glial-like processes extending through the GCL. In addition to the lack of radial glial processes extending through the GCL, note that several GFAP-labeled astrocytes from FPI mice displayed varying levels of activated morphology in the hilus (arrows). In (c), quantitative analysis of the number of radial glial processes extending through the granule cell layer reveals a significant decrease (^*∗*^ *P* < 0.05) in FPI mice compared to sham mice. In (d), the sham micrograph from (a) is shown after thresholding, to depict the technique used for densitometric analysis of GFAP-labeling in the hilus. Once thresholded, the Image J software (NIH v.1.49) automates the counting of white pixels. In (e), the micrograph from the FPI mouse is shown after thresholding. In (f), densitometric analysis of GFAP-labeled astrocytes in the hilus revealed no significant differences between sham and FPI mice at 30 days after FPI. It is pertinent to note that the subgranular zone was excluded from this analysis (as shown in Figure 1) because the cells in this region have a different appearance than they do in the hilus. Scale bars in all images = 30 *μ*m.

Qualitative analysis of the astrocytes at the border between the subgranular zone and the granule cell layer revealed that, compared to sham mice (Figure 3(a)), FPI mice exhibited a hypertrophied appearance and a change in their orientation, such that their processes preferentially extended toward the hilus, rather than towards the molecular layer (Figure 3(b)). As these processes are derived from the GFAP-expressing astrocytes at the border between the subgranular zone and the granule cell layer, we examined the appearance of this population of astrocytes. Quantitative analysis of the number of radial glial-like processes extending through the granule cell layer revealed that, at 30 days after FPI, the number of these processes is significantly decreased in FPI mice compared to sham mice (*t* = 4.919; *P* < 0.05; Figure 3(c)).

### 3.3. Density of GFAP-Labeling in the Hippocampus

The morphology of the GFAP+ astrocytes in the sham mice appeared relatively normal (Figure 3(d)), whereas some of these cells from FPI mice exhibited a moderate hypertrophy compared to the sham mice (Figure 3(e)). As the injury model we use is considered to be mild to moderate, we do not expect a robust hypertrophy to be observed [43], especially at the 30-day post-FPI time point. Despite these morphological alterations, the densitometric analysis of GFAP-labeling in the hilus revealed no significant difference in GFAP-labeling at 30 days after an FPI (*t* = 0.894; *P* = 0.365,  NS; Figure 3(f)). It is pertinent to note that this analysis did not include the border region between the subgranular zone and the granule cell layer because the GFAP+ cells in this region have a distinctly different appearance than those in the hilus.

### 3.4. Basal Dendrites Grow along an Ectopic Glial Scaffold

**Figure 4:**
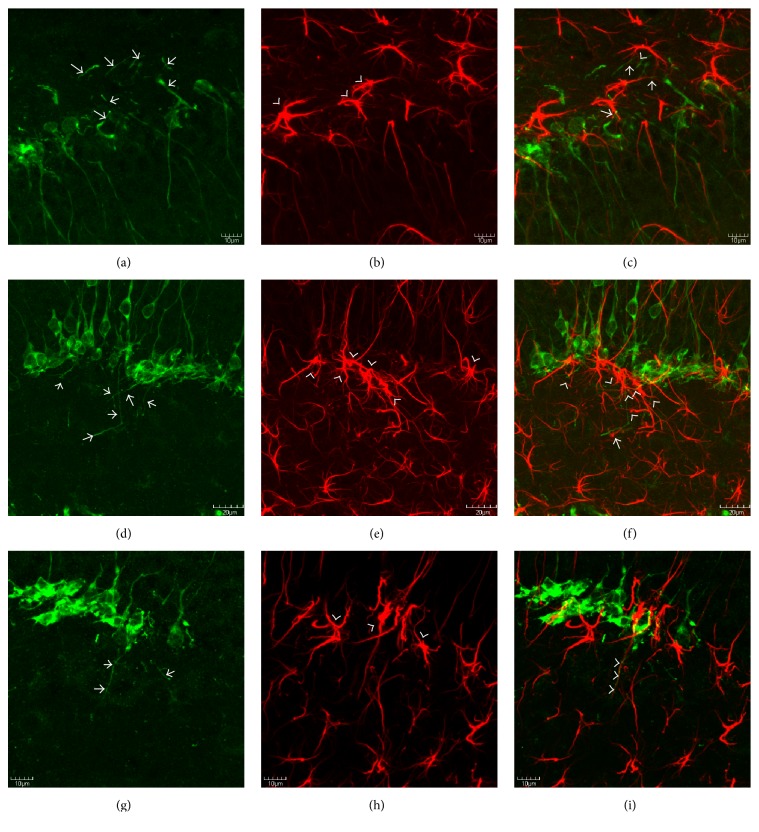
DCX-labeled basal dendrites grow along an ectopic glial scaffold. Confocal micrographs depicting DCX+ (a, d, g), GFAP+ (b, e, h), and merged, double-labeling for DCX (green) and GFAP (red) at 30 days after FPI. Note that (a)–(c) are taken from the infrapyramidal blade, whereas (d)–(i) are taken from the suprapyramidal blade. As can be seen in (a), (d), (g), DCX+ basal dendrites (arrows) are prominent features after FPI, and many of these basal dendrites extend deep into the hilus. Also note, in (b), (e), and (h), the hypertrophied appearance of GFAP+ cells at the base of the granule cell layer (arrowheads). Also note that the predominant orientation of the astrocytes from FPI mice at the border of the granule cell layer is oriented either parallel with the granule cell layer or toward the hilus. Such an orientation is consistent with an ectopic glial scaffold. The lack of radial glial processes extending through the granule cell layer is also notable in (b) and (h). In the merged images (c, f, i) the close relationship between the DCX+ basal dendrites and the GFAP+ processes can be appreciated (arrows). In (i), the apposition of the basal dendrite with the GFAP+ is so close that the two processes almost appear as if they are intertwined (arrowheads). Future studies using electron microscopy will determine if these cells/processes are aberrantly targeted for synaptogenesis in the hilus. Scale bars in (a)–(c) & (g)–(i) = 10 *µ*m and in (d)–(f) = 20 *µ*m. In (f) and (i), the appositions of the basal dendrites to the GFAP+ processes are so close that the two almost appear as if they are intertwined.

Previous studies from pilocarpine-induced epileptic mice revealed that basal dendrites from newborn neurons aberrantly extend into the hilus by growing along the hypertrophied processes of radial glial-like cells. Because the model of FPI used in the current study is known to increase seizure susceptibility [41] and the sprouting of hilar basal dendrites and subsequent synaptic targeting is known to be proepileptic, we sought to determine if a similar anatomical substrate for basal dendrite sprouting was present following an FPI. The results show that basal dendrites from the DCX+ cells extend deep into the hilus at 30 days after FPI (Figures 4(a), 4(d), and 4(g)). Moreover, the results also show the presence of an ectopic glial scaffold that is comprised of the hypertrophied processes of GFAP-labeled astrocytes at the base of the granule cell layer (Figures 4(b), 4(e), and 4(h)). Interestingly, DCX-labeled basal processes from immature granule cells are observed in close apposition to these processes (Figures 4(c), 4(f), and 4(i)).

## 4. Discussion

The results from this study are the first to show ectopic migration of newborn granule cells into hilus, as well as an anatomical substrate for the aberrant growth of newborn neurons following an FPI. These results also indicate that astrocyte hypertrophy may contribute to detrimental neuronal plasticity, because the hypertrophied processes of radial glial-like astrocytes lose many of their radial glial processes and instead preferentially orient toward the hilus (Figure 4). The fact that the DCX+ basal dendrites that extend into the hilus are closely apposed to the GFAP+ processes suggests that the astrocytes provide an ectopic glial scaffold for the aberrant growth of newborn neurons. Therefore, targeting the astrocyte/neuronal interactions might be a novel strategy for normalizing neuronal function and treating the long-term consequences of TBIs.

Neuroinflammation is one of the well-known consequences of TBIs. While the precise mechanisms and positive or negative consequences of the inflammatory cascades that follow are not fully understood, it is known that glial activation and hypertrophy are typical anatomical manifestations of the neuroinflammatory response [11, 12]. Indeed, there are numerous reports of astrocyte and microglial hypertrophy from a number of different models of TBI and from clinical cases of TBI. What is of particular interest in this context is the fact that although injuries do not necessarily have to directly impact the hippocampus, glial activation is still often observed in this region [6, 41, 44, 45]. In this paper, the 30-day timepoint was selected to examine chronic, rather than acute changes to the hippocampus. While the acute changes may be more robust, the time course of development and integration of newborn neurons into the existing hippocampal circuitry is at minimal 2 weeks [46]. Moreover, most of the neurons that are affected during the early stages after brain insult die before they can be functionally integrated into the circuitry [47], as functional integration typically occurs beginning around 4 weeks after the birth of the newborn neurons [48]. Considering the notable role of the hippocampus in a number of cognitive and affective behaviors [44, 49], it is possible that glial activation within the hippocampus contributes to dysfunction of the hippocampal circuitry.

Examples of a role for glial activation in altered hippocampal functioning are observed in the adult neurogenesis literature. Hippocampal neurogenesis is ongoing throughout the lifespan of many mammals, including rodents, nonhuman primates, and humans [50–52]. A number of studies have documented the importance of adult neurogenesis in cognitive, affective, and reproductive behaviors [53–55]. In many of these studies, alterations to normal hippocampal neurogenesis result in varying degrees of functional alterations to behavioral and cognitive outcomes [56]. Our finding that the processes of hypertrophied astrocytes provide an ectopic glial scaffold (Figure 4) for the aberrant growth and/or migration of immature dentate granule cells represents one mechanism through which glial cells can contribute to altered hippocampal functioning.

Interestingly, previous studies have indicated that aberrantly sprouted basal dendrites from immature granule cells can be targeted for synaptogenesis in the hilus [13, 16]. A previous report showed that, following a controlled cortical impact TBI, ectopic migration and dendritic branching of newborn dentate granule cells occur [57]. These newborn neurons were observed to be functionally integrated into the hippocampal circuitry and their potential for maladaptive plasticity was noted [57]. Despite the fact that we did not observe a difference in the number of DCX-labeled cells at the 30 days' post-FPI time point, the appearance of aberrant sprouting and ectopic migration suggests that neurogenesis is chronically altered after FPI. It is also possible that if we examine later time points, neurogenesis could significantly decrease. Thus, there are several mechanisms through which hypertrophied glial cells can directly influence hippocampal circuitry, and altered hippocampal circuitry can underlie functional deficits including increased seizure susceptibility and the development of posttraumatic epilepsy [58, 59].

Posttraumatic epilepsy (PTE) is the most common type of acquired epilepsy [60]. A number of mechanisms have been postulated to contribute to the development of PTE. These include oxidative and metabolic changes/dysfunction [61], ion channel alterations [62], inflammation [63, 64], synaptic plasticity [65], loss and/or reduced efficacy of inhibitory interneurons [66], and a number of other possibilities [67, 68]. Parallels can be drawn with the pilocarpine model of temporal lobe epilepsy in which an initial chemotactic, proconvulsive insult leads to status epilepticus. The status epilepticus is inhibited using benzodiazepines or other sedatives [69], after which a latent period of >2 weeks typically occurs with little to no seizure activity. Eventually, most, if not all, of these rodents will go on to develop spontaneous epileptiform discharges that is analogous to temporal lobe epilepsy. In the pilocarpine model, as in our current study, hilar basal dendrite sprouting from immature granule cells is a prominent feature [13]. Of further interest is that, in the pilocarpine model, the hypertrophied astrocytes at the base of the granule cell layer were observed to express CCR2, which is the receptor for monocyte chemoattractant protein (MCP-1), also known as CCL2 [14]. Signaling through the CCR2/CCL2 receptor/ligand has been shown to be involved in chemotactic guidance of neuroblast migration [70–72]. Considering that we have previously demonstrated an increase of CCL2 protein in our FPI model [5, 6], it is possible that this mechanism is involved in the aberrant sprouting of basal dendrites from the newborn neurons, as well as the ectopic migration of some of these granule cells into the hilus (Figure 1). Future studies are warranted to examine this possibility

In conclusion, we demonstrate, for the first time, that radial glial fibers from GFAP+ astrocytes at the base of the granule cell layer are chronically reduced following FPI and that the hypertrophied astrocytes in this region can provide an anatomical substrate for the aberrant growth and migration of immature neurons in the hippocampus. Based on previous evidence of altered hippocampal function in response to such anatomical changes, future studies are needed to determine the potential of these alterations as targets for therapeutic intervention after TBI. Understanding these mechanisms could also lead to more efficacious regenerative therapy approaches.
